# Insurance-Based Disparities in Congenital Cardiac Operations in the Era of the Affordable Care Act

**DOI:** 10.1007/s00246-023-03136-7

**Published:** 2023-03-12

**Authors:** Catherine G. Williamson, Mina G. Park, Bailey Mooney, Aditya Mantha, Arjun Verma, Peyman Benharash

**Affiliations:** 1 Cardiovascular Outcomes Research Laboratories (CORELAB), Division of Cardiac Surgery, David Geffen School of Medicine at UCLA, 10833 Le Conte Avenue, 64-249 CHS, Los Angeles, CA 90095 USA; 2 Division of Cardiology, University of Southern California Keck School of Medicine, Los Angeles, CA USA

**Keywords:** Congenital Cardiac Surgery, Costs, Readmissions

## Abstract

A body of literature has previously highlighted the impact of health insurance on observed disparities in congenital cardiac operations. With aims of improving access to healthcare for all patients, the Affordable Care Act (ACA) expanded Medicaid coverage to nearly all eligible children in 2010. Therefore, the present population-based study aimed to examine the association of Medicaid coverage with clinical and financial outcomes in the era the ACA. Records for pediatric patients (≤ 18 years) who underwent congenital cardiac operations were abstracted from the 2010–2018 Nationwide Readmissions Database. Operations were stratified using the Society of Thoracic Surgeons-European Association for Cardio-Thoracic Surgery (STAT) Category. Multivariable regression models were developed to evaluate the association of insurance status on index mortality, 30-day readmissions, care fragmentation, and cumulative costs. Of an estimated 132,745 hospitalizations for congenital cardiac surgery from 2010 to 2018, 74,925 (56.4%) were insured by Medicaid. The proportion of Medicaid patients increased from 57.6 to 60.8% during the study period. On adjusted analysis, patients with Medicaid insurance were at an increased odds of mortality (1.35, 95%CI: 1.13–1.60) and 30-day unplanned readmission (1.12, 95%CI: 1.01–1.25), experienced longer lengths of stay (+ 6.5 days, 95%CI 3.7–9.3), and exhibited higher cumulative hospitalization costs (+ $21,600, 95%CI: $11,500–31,700). The total hospitalization cost-burden for patients with Medicaid and private insurance were $12.6 billion and $8.06 billion, respectively. Medicaid patients exhibited increased mortality, readmissions, care fragmentation, and costs compared to those with private insurance. Our results of outcome variation by insurance status indicate the necessity of policy changes to attempt to approach equality in surgical out comes for this high-risk cohort.

**Graphical Abstract:**

Baseline characteristics, trends, and outcomes by insurance status over the ACA rollout period 2010–2018

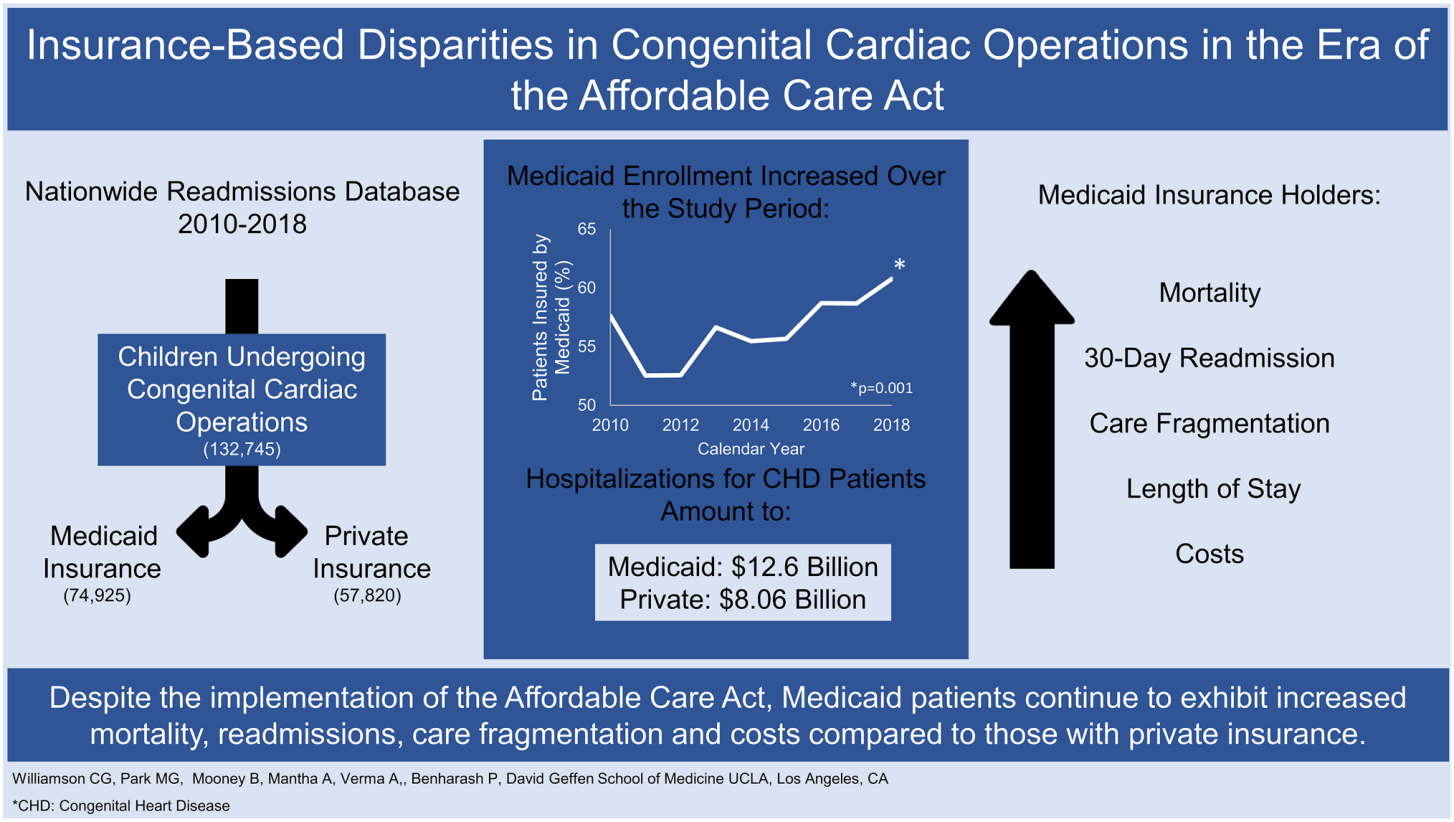

**Supplementary Information:**

The online version contains supplementary material available at 10.1007/s00246-023-03136-7.

## Introduction

Present in 1% of the United States population, congenital heart disease (CHD) encompasses a wide spectrum of defects, and in nearly quarter of cases requires surgical intervention within the first year of life [1]. Despite significantly improved overall long-term survival in patients with CHD, socioeconomic disparities persist. A large body of literature has demonstrated public insurance to be reliably associated worse clinical outcomes among patients requiring surgical correction for CHD [2–4].These studies exist within a growing awareness of systemic barriers to healthcare access among disadvantaged groups across the United States [5, 6].

The Affordable Care Act (ACA) of 2010 offered a possible solution by providing affordable insurance coverage for over 50 million uninsured Americans [5]. The bill included specific provisions to guarantee coverage for children despite pre-existing conditions while reducing out-of-pocket costs [7]. Nonetheless, some have questioned the pragmatic impact of ACA on quality of care, especially in light of its inconsistent roll-out across the US [5]. Interestingly, Medicaid coverage itself, has been associated with poor longitudinal care, in-hospital complications and resource use following several categories of noncardiac operations [8]. These differences have, in part, been attributed to inconsistencies in referral patterns that affect timely diagnosis and evaluation, access to quality care, and postoperative follow-up. Patients with public insurance may also experience delays in referral for surgical therapy of congenital heart disease [9, 10]. However, data regarding insurance-based disparities in outcomes of CHD operations in the era of ACA is lacking. The present work aimed to examine the association of Medicaid coverage with financial and clinical outcomes of operations in children with CHD in a nationally representative database. These endpoints included mortality, costs, length of stay, discharge disposition, urgent readmissions, and care fragmentation. We hypothesized Medicaid insurance to be associated with increased risk of adverse outcomes compared to those with private insurance.

## Methods

All pediatric (≤ 18 years) patients undergoing congenital cardiac operations were tabulated in the 2010–2018 Nationwide Readmissions Database (NRD). The NRD is the largest all-payer inpatient and readmission database that is maintained as part of the Healthcare Cost and Utilization Project [11]. The database uses hospital specific based discharge weights to provide accurate national estimates for 36 million discharges annually. Using hospital-specific weighting algorithms, the NRD is able to accurately estimate 58% of US hospitalizations. For patients under the age of one, the NRD can estimate approximately 50% of hospitalizations [11].

The *International Classification of Disease Ninth and Tenth Revision* procedure and diagnosis codes (ICD-9 and ICD-10) were utilized to classify operations of interest. These included operative repair for ventricular/atrial septal defects, patent ductus arteriosus, Tetralogy of Fallot, arterial switch procedure, Norwood procedure, Glenn procedure, Fontan operation, truncus arteriosus repair, coarctation repair, and total anomalous venous pulmonary return repair, as has been previously described [12–14]. All operations were cross-validated to ensure appropriate clinical diagnosis codes. The Society of Thoracic Surgeons-European Association for Cardio-Thoracic Surgery (STAT) Category, a previously validated stratification tool for mortality risk following congenital operations, was used to account for operative complexity [15]. Pre-populated variables including insurance status, median household income, age, and mortality were defined according to the NRD data dictionary [16]. Hospitalizations with missing data or those without private or Medicaid insurance were excluded (1.5%). Other clinical characteristics were defined using ICD9/10 codes as reported in prior work [17]. The Charlson Comorbidity Index, a validated composite of comorbid conditions [18], was utilized to quantify the burden of comorbidities. This index is a standardized, 10-point measure of patient comorbidities which includes neurologic, cardiac, vascular, endocrine, pulmonary, renal, hepatic, gastrointestinal, cancer, and immune categories. Pediatric comorbidities were defined using previously published diagnosis groups [14]. To assess annual volume status for each center, congenital cardiac case volume was summed each year while accounting for appropriate survey weights. Hospitals were classified as low, medium, or high-volume based on tertiles of annual case volume with cutoffs at the 33rd and 66th percentile.

Outcomes of interest included mortality during the index hospitalization, 30-day non-elective readmission, and inpatient costs of care. Elective readmissions were not included in the readmissions analysis to exclude scheduled follow-up. Discharge disposition, length of stay, and care fragmentation were also examined. As NRD tracks patients within one calendar year, discharges in December of each year were excluded to allow adequate follow-up time. Patients returning to a hospital on readmission that was not the same center as the index hospitalization were considered to experience care fragmentation [19]. Hospitalization costs were calculated using specific hospital NRD cost-to-charge ratios and were normalized to the 2018 Personal Health Care Index. Within this analysis, categorical variables are reported as frequencies (%) and normally distributed continuous variables as means with standard deviations (SD). Non-normally distributed continuous factors are reported with medians and interquartile ranges (IQR). To assess significance of differences, we utilized the Pearson’s chi-square test and the adjusted Wald test for categorical and continuous factors, respectively. Standardized mean differences were obtained by dividing the mean by the standard deviation to demonstrate effect size [20]. Cuzick’s non-parametric test was utilized to examine the significance of temporal trends (*nptrend*) [21].

Multivariable linear and logistic regression models were developed to evaluate the independent association of insurance status with outcomes such as mortality, readmissions, and costs. Models adjusted for factors including age-group, gender, operative type, patient comorbidities, STAT Category, operative volume, Charlson Comorbidity Index, and calendar year (Supplemental Tables 1–3). Model covariates were selected using the Elastic Net regularization method [22]. This algorithm uses a penalized least squares methodology to select model covariates to reduce collinearity of covariates and overfitting. In order to both generate and test the model; the study cohort was divided into equal (1:1) derivation and validation cohorts. Three iterations of tenfold cross-validation were performed over alpha values of 0.5, 0.75, and 1.0. After retention of clinically relevant variables, the final model was selected using receiver operating characteristic as well as Akaike and Bayesian information criteria, when appropriate. Models were verified using entropy balancing; a novel reweighting technique to ensure matched samples (Supplemental Table 4) [23]. The risk adjusted association of covariates with specified outcomes are reported as adjusted odds ratios (AOR) for dichotomous outcomes and beta coefficients (β) for continuous variables, with 95% confidence intervals (95% CI) for both. The Stata *margins* command was used to predict absolute risk adjusted values for various outcomes based on the output of relevant regressions.

Survival analysis of 30-day readmission was subsequently examined using Royston-Parmar flexible parametric regression [24]. This methodology was selected due to the variable hazards of readmission over the selected 30-day time period. The hazards were calculated over days to readmission using two restricted cubic spline knots. An α < 0.05 was considered statistically significant for all analyses. All analyses were preformed using Stata 16.1. This study was deemed exempt from full review by the Institutional Review Board at the University of California, Los Angeles. IRB#17–001112. Approved: 7/26/2017.

## Results

### Patient Characteristics

Of an estimated 132,745 hospitalizations for congenital cardiac surgery, 74,925 (56.4%) were insured by Medicaid. Before survey weighting, the Medicaid group compromised 16,327 (56.3%) of 29,018 total admissions. Over the study period, the proportion of Medicaid patients significantly increased from 57.6 to 60.8% of the entire cohort (*nptrend* = 0.001). Compared to those with private insurance, patients with Medicaid coverage had a higher burden of comorbidities as measured by the Charlson Comorbidity Index (0.40 vs 0.33, *p* < 0.001), were more frequently within the lowest median income quartile (35.9 vs 15.8%, *p* < 0.001) and were comprised of similar proportions of neonatal patients (21.1 vs 21.9%, *p* = 0.52, Table 1). Medicaid patients were also more likely to suffer from a congenital kidney disorder (4.6 vs 3.6%, *p* = 0.001) and pulmonary hypertension (6.6 vs 5.1%, *p* < 0.001). While the median STAT Category was similar between groups (2 (1–3) vs 2 (1–3), *p* = 0.86), Medicaid patients comprised higher proportions of Fontan operations (6.2 vs 5.1%, *p* = 0.003) or repairs of total anomalous pulmonary venous return (3.7 vs 2.5%, p < 0.001) but lower proportions of arterial switch procedures (3.6 vs 5.1%, *p* < 0.001) or coarctation of aorta repairs (6.6 vs 7.9%, *p* = 0.006, Table 1). Of note, the proportion of Medicaid patients treated at a high-volume center was lower than that for those insured privately (91.3 vs 92.4%, *p* = 0.04, Table 1).

**Table 1:** Comparison of baseline patient characteristics by insurance status

	Private (*n* = 57,820)	Medicaid (*n* = 74,925)	*p* value	SMD
Demographics (No. %)				
Elective Admission	34,769 (60.2)	42,819 (57.2)	0.01	0.07
Charlson Comorbidity Index (points, IQR)	0.33 (0–1)	0.40 (0–1)	< 0.001	0.07
Female Sex	26,535 (45.9)	35,576 (47.5)	0.06	0.03
Age Group (No. %)				
Neonate	12,663 (21.9)	15,809 (21.1)	0.52	0.002
Infant	30,992 (53.6)	41,883 (55.9)	0.005	0.07
1–3 Years	4,683 (8.1)	6,593 (8.8)	0.002	0.08
4–10 Years	5,843 (10.1)	7,863 (10.5)	0.78	0.06
11–18 Years	3,603 (6.2)	2,797 (3.7)	0.02	0.10
Median Income Quartile (No. %)			< 0.001	0.68
0–24^th^	9,034 (15.8)	26,537 (35.9)		
25–49^th^	13,514 (23.6)	22,293 (30.1)		
50–74^th^	17,574 (30.7)	17,013 (23.0)		
75–99^th^	17,043 (29.8)	8,069 (10.9)		
Medical Conditions (No. %)				
Chromosomal Abnormality	6,969 (12.1)	9,280 (12.4)	0.59	0.02
Congenital Kidney Disorder	2,108 (3.6)	3,464 (4.6)	0.001	0.05
Congenital Respiratory Disorder	1,911 (3.3)	3,020 (4.0)	0.007	0.04
Congenital Nervous Disorder	1,133 (2.0)	1,762 (2.4)	0.08	0.04
Pulmonary Hypertension	2,922 (5.1)	4,965 (6.6)	< 0.001	0.06
Procedure Type (No. %)				
Glenn Procedure	3,175 (5.5)	5,036 (6.7)	0.11	0.07
Norwood Procedure	7,342 (12.7)	9,656 (12.9)	0.82	0.02
Fontan Operation	2,943 (5.1)	4,625 (6.2)	0.003	0.06
Tetralogy of Fallot Repair	4,652 (8.0)	5,655 (7.5)	0.50	0.04
Total Anomalous Pulmonary Venous Return Repair	1,459 (2.5)	2,747 (3.7)	< 0.001	0.05
Truncus Arteriosus Repair	347 (0.6)	565 (0.8)	0.24	0.01
Arterial Switch	2,958 (5.1)	2,665 (3.6)	< 0.001	0.07
Coarctation of Aorta Repair	4,567 (7.9)	4,937 (6.6)	0.006	0.07
Atrial Septal Defect Repair	17,317 (29.9)	19,153 (25.6)	< 0.001	0.10
Ventricular Septal Defect Repair	13,366 (23.1)	16,999 (22.7)	0.75	0.01
Patent Ductus Arteriosus Repair	21,691 (37.5)	29,654 (39.6)	0.01	0.06
STAT Category (points, IQR)	2 (1–3)	2 (1–3)	0.86	0.01
Hospital Teaching Status (No. %)			0.08	0.02
Urban Non-teaching	1,360 (2.4)	2,083 (2.8)		
Urban Teaching	56,357 (97.4)	72,747 (97.1)		
Rural	102 (0.2)	95 (0.1)		
Operative Volume (No. %)			0.04	0.05
Low	1,041 (1.8)	1,124 (1.5)		
Medium	3,353 (5.8)	5,394 (7.2)		
High	53,416 (92.4)	68,393 (91.3)		

*STAT* Society of Thoracic Surgeons-European Association for Cardio-Thoracic Surgery, *SMD* Standardized mean Difference, *IQR* Interquartile Range

### Univariate Outcomes

**Fig. 1:**
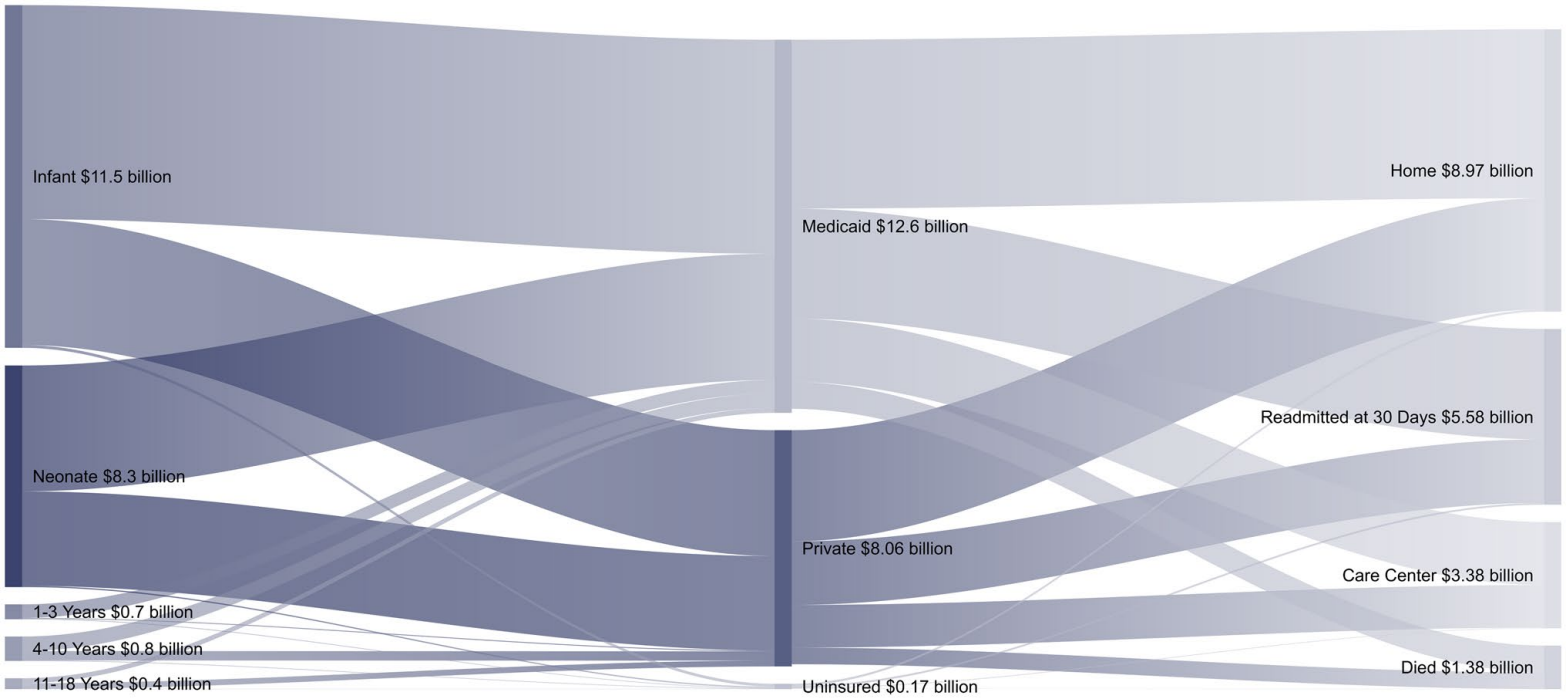
Total Index Hospitalization Costs of Congenital Cardiac Operations by Insurance and Outcome. Costs are cumulative over the study period by age group, insurance status, and discharge disposition. Costs shown in $1,000,000 USD

A bivariate comparison of outcomes during the index hospitalization are shown in Table 2. Patients insured by Medicaid suffered higher rates of mortality (2.6 vs 2.0%, *p* = 0.04), infection (4.0 vs 2.9%, *p* = 0.008), and sepsis (2.7 vs 1.7%, *p* = 0.005, Table 2). Furthermore, these patients more commonly required prolonged mechanical ventilation (> 96 h) (35.6 vs 30.9%, *p* < 0.001) and gastrostomy tube placement (3.9 vs 2.5%, p < 0.001), compared to those on private insurance (Table 2). Patients with Medicaid insurance experienced higher rates of non-home discharge (16.9 vs 15.1%, **p** = 0.02) and longer median lengths of stay 8 (4–23) vs 7 (4–16) days, p < 0.001). Medicaid patients also had higher median hospitalization costs per patient ($57,000 (33,300–125,400) vs 51,100 (31,100–101,600), *p* < 0.001, Table 2). Over the study period, total hospitalization costs for patients with Medicaid amounted to $12.6 billion and those insured privately amounted to $8.06 billion (Fig. 1). Of note, the total hospitalization costs for those readmitted within 30 days was $5.58 billion, 26.8% of the total cost burden, while representing only 14.4% of patients. Following discharge, rates of non-elective 30-day readmission (15.3 vs 14.0%, *p* = 0.04), and care fragmentation (10.9 vs 7.2%, *p* < 0.001) were significantly higher among those with Medicaid coverage (Table 2).

**Table 2:** Unadjusted patient outcomes stratified by insurance status

	Private (*n* = 57,820)	Medicaid (*n* = 74,925)	*p* value	SMD
Complication (No. %)				
Mortality	1,169 (2.0)	1,950 (2.6)	0.04	0.05
Acute Kidney Injury	2,814 (4.9)	4,077 (5.4)	0.15	0.03
Hemorrhage	2,679 (4.6)	3,498 (4.7)	0.94	0.002
Pneumonia	1,847 (3.2)	2,968 (4.0)	0.07	0.01
Infection	1,691 (2.9)	2,971 (4.0)	0.008	0.03
Sepsis	978 (1.7)	2,001 (2.7)	0.005	0.05
Interventions (No. %)				
Extracorporeal Life Support	1,412 (2.4)	2,258 (3.0)	0.14	0.04
Prolonged Mechanical Ventilation	17,890 (30.9)	26,670 (35.6)	< 0.001	0.08
Gastrostomy Tube	1,423 (2.5)	2,950 (4.0)	< 0.001	0.08
Resource Utilization				
Non-Home Discharge (No. %)	8,739 (15.1)	12,691 (16.9)	0.02	0.08
30-Day Readmission (No. %)	7,426 (14.0)	10,550 (15.3)	0.04	0.05
Care Fragmentation (No. %)	4,163 (7.2)	8,167 (10.9)	< 0.001	0.08
Length of Stay (days, IQR)	7 (4–16)	8 (4–23)	< 0.001	0.15
Index Cost ($1,000 USD, IQR)	51.1 (31.1–101.6)	57.0 (33.3–125.4)	< 0.001	0.10
Log-Transformed Costs (SD)	11.2 ± 0.96	11.3 ± 1.01	< 0.001	0.12
Readmission Cost ($1,000 USD, IQR)	55.1 (13.1–125.2)	46.0 (11.5–143.8)	0.19	0.05
Cumulative Costs ($1,000 USD, IQR)	166.9 (88.5–318.1)	172.7 (85.4–359.0)	< 0.001	0.10

*IQR* Interquartile Range, *SMD* Standardized mean Difference, *SD* Standard Deviation

### Trends

While the observed risk of mortality decreased for the overall study population (2.9–1.9%, *nptrend* = 0.02) and among those with private insurance (2.7–1.4%, *nptrend* = 0.03) during the study period, patients covered by Medicaid did not experience a significant decline in risk for death (3.2–2.2%, *nptrend* = 0.08). Rates of 30-day unplanned readmission did not significantly change over the study period (13.4–16.2%, *nptrend* = 0.13).

### Risk Adjusted Outcomes

**Fig. 2:**
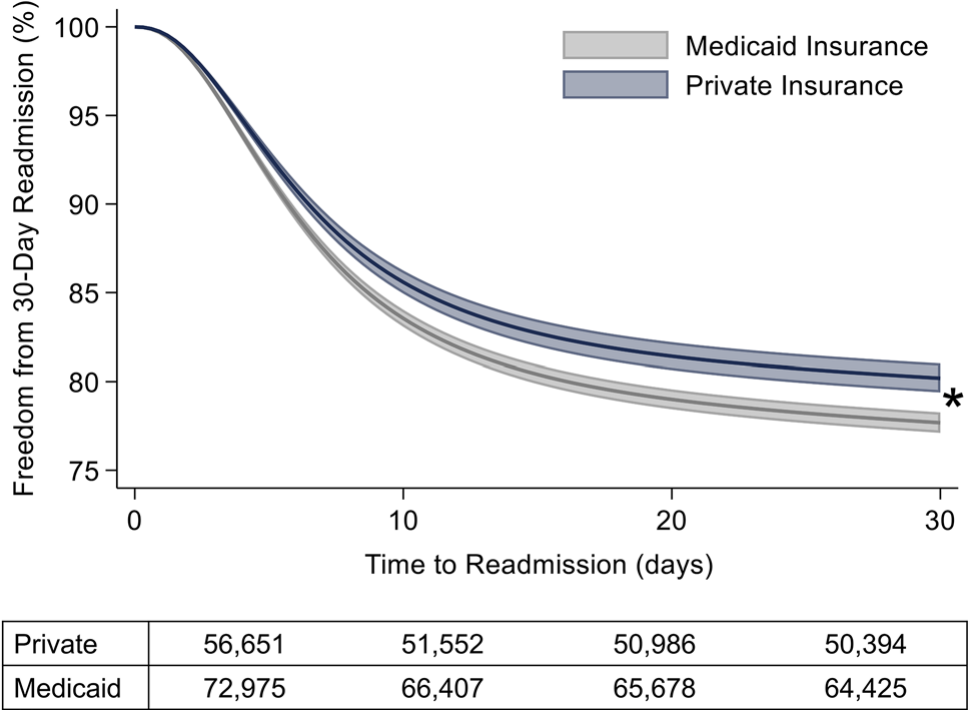
Royston Parmar Adjusted Model of 30-Day Unplanned Readmission by Insurance Status. Multivariable adjusted regression model of freedom from readmission over 30 days post-discharge, with higher risk of readmissions corresponding to decreased freedom from readmission. Model shown with 95% confidence intervals. Model adjusted for age, gender, congenital operation performed, patient comorbidities, STAT Category, operative volume, Charlson Comorbidity Index, and calendar year

**Fig. 3:**
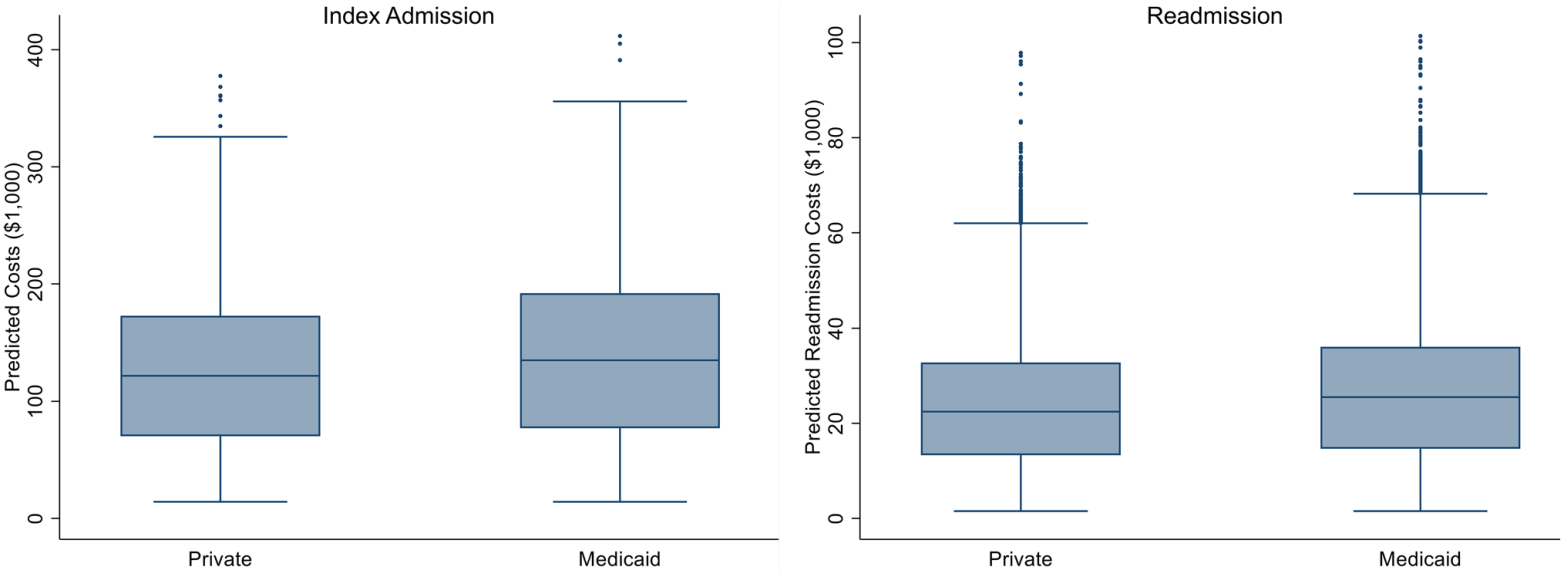
Adjusted Hospitalization Costs of Index and Re-admissions for Congenital Cardiac Patients by Insurance Status. Multivariable adjusted costs per patient by insurance status. Model adjusted for age, gender, congenital operation performed, patient comorbidities, STAT Category, operative volume, Charlson Comorbidity Index, and calendar year. The midline indicates the median value, with the boxes indicating 25th-75th percentile. The bars show all values within the sample, with the exception of outliers, shown as dots

On adjusted analysis and compared to those insured privately, patients with Medicaid insurance were at an increased adjusted odds of index mortality (AOR: 1.35, 95% CI: 1.13–1.60), 30-day unplanned readmission (AOR: 1.12, 95% CI: 1.01–1.25), non-home discharge (AOR: 1.14, 95% CI: 1.02–1.29), and care fragmentation (AOR: 1.70, 95% CI: 1.18–2.45, Table 3). As shown in Fig. 2, the increased odds of readmission were further supported with a Royston Parmar flexible parametric model. Furthermore, Medicaid patients experienced incremental increases in length of stay (+ 6.5 days, 95% CI: 3.7–9.3), index hospitalization costs (+ $16,300, 95% CI: 8,500–24,200, Fig. 3), readmission costs (+ $5,300, 95% CI: 1,100–9,500, Fig. 3), and total hospitalization costs per patient (+ $21,600, 95% CI: 11,500–31,700,).

**Table 3:** Risk-adjusted impact of Medicaid insurance on outcomes

	AOR	95% CI	*p* value
Mortality	1.35	1.13–1.60	< 0.001
Non-Home Discharge	1.14	1.02–1.29	0.02
30-Day Readmission	1.12	1.01–1.25	0.03
Care Fragmentation	1.70	1.18–2.45	0.005

	*β* Coefficient	95% CI	*p* value
Length of Stay (days)	+ 6.5	3.7–9.3	< 0.001
Index Cost ($1,000 USD)	+ 16.3	8.5–24.2	< 0.001
Readmission Cost ($1,000 USD)	+ 5.3	1.1–9.5	0.01
Total Cumulative Costs ($1,000 USD)	+ 21.6	11.5–31.7	< 0.001

95% CI 95% Confidence Interval, *AOR* Adjusted Odds Ratio, *β* Beta Coefficient, Reference: Private Insurance

## Discussion

In this population-based study of children with CHD following enactment of ACA, we evaluated the impact of Medicaid coverage on clinical and financial outcomes of surgical treatment. Despite previously reported variable adoption of ACA across the US, the proportion of patients covered by Medicaid increased from 57.6% in 2010 to 60.8% in 2018. Medicaid insurance was associated with stagnant risk of mortality and generally worse in-hospital outcomes. With overall higher costs of care, Medicaid patients also faced increased odds of unplanned readmission and care fragmentation. Several of our findings warrant further discussion.

### Trends Over the Study Period

Our study encompassed the period following the introduction of the ACA across the US. Since its initial provision of this legislation in 2010, the proportion of patients insured by Medicaid increased significantly. While overall mortality decreased in the same time period, this was not the case among Medicaid recipients. The overall reduction in mortality may be attributable to improved screening, advancements in medical technology and surgical techniques as well as perioperative management of patients with CHD [25, 26]. A prior international study noted improved survival in these patients despite increasing case-complexity from 2000 to 2010. [25]. Further, in their analysis of state-implemented newborn screening, Abouk et al. found that mandatory policies for critical congenital heart disease to be associated with significant reduction in infant cardiac death between 2007 and 2013 [26]. However, these benefits may be isolated to patients with consistent access to high-quality care. It is possible that Medicaid patients may have decreased ability to cross state lines or gain referrals to congenital centers of excellence at the same rate as those with private insurance [27, 28]. Further, in our study, Medicaid patients had increased burden of disease yet lower prevalence at high-volume centers. This factor may promote the notion that patients are presenting at later disease points or are experiencing delayed referrals to high-quality care [29–31]. As Medicaid patients continue to seek out affordable care [32], the continued impact of these policies may become realized, emphasizing the need for future studies assessing outcomes for pediatric CHD patients by insurance status.

### Clinical Outcomes by Insurance Status

Despite improved access to healthcare and decreasing mortality rates overall, CHD patients primarily covered by Medicaid exhibited increased adjusted odds of in-hospital mortality in comparison to those insured privately. Medicaid coverage was also associated with prolonged hospitalization duration, and higher odds of non-home discharge, 30-day readmission, and care fragmentation. The inferior clinical outcomes noted here may indicate an inequity in access to high-performing centers, with insurance type previously shown to influence both referral patterns and perioperative outcomes of CHD [2, 3, 33]. Using a national cohort of cancer patients, Nabi and colleagues showed that access to high-volume centers was improved among Medicaid patients following enactment of the ACA, but that barriers in access to care persisted during that time period [34]. Our findings of inferior clinical outcomes for Medicaid patients may represent a similar access disparity, manifesting as increased patient mortality and morbidity for children with CHD. Moreover, Medicaid coverage may limit out of state access for patients, regardless of illness severity [27, 28]. Cunningham et al. postulated that despite providing more governmental funding for hospitalizations, the ACA policies also may have concentrated un- and under-insured patients to safety-net centers [32]. Already strained, safety net hospitals may be particularly affected by performance penalties under ACA decreasing available funds to invest in perioperative care for vulnerable patients [35–37]. However, it is important to note that Medicaid insurance may be surrogate marker for other patient risk factors such as nutrition, access to medications, and poor living conditions. Moreover, this post-ACA Medicaid cohort may now contain a broader range of illness severity due to offering insurance to the previously uninsured, increasing the medical and surgical complexity of this population. Yet, an argument may still be made for further centralization of care for these patients, to ensure minimal numbers of high-risk procedures occurring at low-volume centers. Thus, access to appropriate facilities and longitudinal care, including prenatal monitoring and maternal education [38] are important facets to consider in future quality improvement efforts.

### Financial Outcomes by Insurance Status

With recent emphasis on value-based care beyond episodic hospitalizations, discharge planning and adequate follow-up have garnered much attention. These initiatives have been shown to improve unplanned readmissions, patient mortality, and costs of care [39]. In our study, Medicaid patients exhibited higher rates of unplanned readmission and costs during initial and rehospitalizations compared to those insured privately. Furthermore, total hospitalization costs for patients requiring urgent readmission amounted to almost $6 billion over the study period, a disproportionally large portion of healthcare spending for surgical management of CHD. The excess expenditures may in part be attributable to higher rates of complications and unplanned readmissions 39, 40. Importantly, Lawson and colleagues reported that preventing postoperative complications in Medicare-insured adults could reduce urgent readmissions by 40,000 patients per year, saving an annual $620 million in the US [41]. This observation is particularly relevant since 42.6% of healthcare expenditures now support public insurance programs including Medicare and Medicaid [26]. Therefore, investment in high-quality care for patients with CHD may paradoxically decrease the overall cost burden of this population by limiting complications and urgent readmissions.

## Limitations

The present study has several important limitations inherent to its retrospective and coding-based nature. Furthermore, the database does not contain anatomic or physiologic information to assess disease severity. The database solely supplies insurance information regarding the primary expected payer, therefore those with dual insurances may be falsely classified in the Medicaid or Private insurance groups. Complications and comorbidities may overlap, as diagnoses are coded across the entire admission, rather than pre- or post-operatively. The Charlson Comorbidity Index was included due to its availability in STATA, but may be inferior to new scores such as the Pediatric Comorbidity Index. Of note, care fragmentation may not always represent a poor outcome, as minor post-operative concerns may be appropriately managed at small, outside hospitals within geographic proximity to the patient. Moreover, we were unable to discern levels of preoperative outpatient care or care coordination. We were similarly unable to account for state-level variation of ACA rollout and its outcomes, as the NRD lacks identification of individual states. Importantly, we do not have data to compare access prior to the ACA enactment prior to 2010 to fully assess if improvements have been made. Finally, the NRD tracks 50% of newborns, but 58% of those over one-year, which may affect weighting mechanisms for comparisons between groups. Despite these limitations, we used the largest available readmissions database along in order to assess the clinical and financial outcomes for patients with CHD in the era of the ACA.

## Conclusion

In conclusion, we have demonstrated inferior clinical and financial outcomes associated with Medicaid insurance at the national level compared to those privately insured. Efforts to improve access to care and referrals to high-performing centers are crucial to providing equitable healthcare for this high-risk population, while simultaneously decreasing healthcare resource utilization. Systemic strategies aimed at enhancing referral of Medicaid beneficiaries to expert centers and improving post-discharge care coordination may advance outcomes in this high-risk population.

## Supplementary Information

Below is the link to the electronic supplementary material.

Supplementary file1 (DOCX 31 KB)

## Abbreviations

ACA: Affordable Care Act
AOR: Adjusted Odds Ratio
β: Beta Coefficient
CHD: Congenital Heart Disease
ICD: International Classification of Diseases
IQR: Interquartile Range
*Nptrend*: Non-Parametric Trend
NRD: Nationwide Readmissions Database
SD: Standard Deviation
STAT: Society of Thoracic Surgeons-European Association for Cardio-Thoracic Surgery
95% CI: 95% Confidence Interval

## References

[1] Data and Statistics on Congenital Heart Defects | CDC. https://www.cdc.gov/ncbddd/heartdefects/data.html. Accessed August 31, 2021.

[2] DeMone JA, Gonzalez PC, Gauvreau K, Piercey GE, Jenkins KJ (2003). Risk of death for medicaid recipients undergoing congenital heart surgery. Pediatr Cardiol.

[3] Chan T, Pinto NM, Bratton SL (2012). Racial and insurance disparities in hospital mortality for children undergoing congenital heart surgery. Pediatr Cardiol.

[4] Peiris V, Singh TP, Tworetzky W, Chong EC, Gauvreau K, Brown DW (2009). Association of socioeconomic position and medical insurance with fetal diagnosis of critical congenital heart disease. Circ Cardiovasc Qual Outcomes.

[5] Kominski GF, Nonzee NJ, Sorensen A (2017). The affordable care act’s impacts on access to insurance and health care for low-income populations. Annu Rev Public Health.

[6] Anderson BR, Fieldston ES, Newburger JW, Bacha EA, Glied SA (2018). Disparities in outcomes and resource use after hospitalization for cardiac surgery by neighborhood income. Pediatrics.

[7] Wisk LE, Gangnon R, Vanness DJ, Galbraith AA, Mullahy J, Witt WP (2014). Development of a novel, objective measure of health care-related financial burden for US. Families with children. Health Serv Res..

[8] Claflin J, Dimick JB, Campbell DA, Englesbe MJ, Sheetz KH (2019). Understanding disparities in surgical outcomes for medicaid beneficiaries. World J Surg.

[9] Perlstein MA, Goldberg SJ, Meaney FJ, Davis MF, Kluger CZ (1997). Factors influencing age at referral of children with congenital heart disease. Arch Pediatr Adolesc Med.

[10] Chang RKR, Rodriguez S, Lee M, Klitzner TS (2006). Risk factors for deaths occurring within 30 days and 1 year after hospital discharge for cardiac surgery among pediatric patients. Am Heart J.

[11] HCUP Nationwide Readmissions Database (NRD). Healthcare Cost and Utilization Project (HCUP). 2014, 2016, and 2017. Agency for Healthcare Research and Quality, Rockville, MD. www.hcup-us.ahrq.gov/nrdoverview.jsp

[12] Jacobs JP, Mayer JE, Mavroudis C (2017). The society of thoracic surgeons congenital heart surgery database: 2017 update on outcomes and quality. Ann Thorac Surg.

[13] Desai J, Aggarwal S, Lipshultz S (2017). Surgical interventions in infants born preterm with congenital heart defects: an analysis of the kids’ inpatient database. J Pediatr.

[14] Williamson CG, Tran Z, Kim S, Hadaya J, Biniwale R, Benharash P (2021). Center volume impacts readmissions and mortality following congenital cardiac surgery. J Pediatr.

[15] Jacobs ML, Pasquali SK, Jacobs JP, O)brien SM. Empirically based tools for analyzing mortality and morbidity associated with congenital heart surgery. In: *Pediatric and Congenital Cardiac Care: Volume 1: Outcomes Analysis*. Vol 1. Springer-Verlag London Ltd; 2015:363–375. doi:10.1007/978-1-4471-6587-3_28

[16] HCUP Databases. Healthcare Cost and Utilization Project (HCUP). 2006–2009. Agency for Healthcare Research and Quality, Rockville, MD. www.hcup-us.ahrq.gov/databases.jsp. 21413206

[17] Sanaiha Y, Khoubian JJ, Williamson CG (2020). Trends in mortality and costs of pediatric extracorporeal life support. Pediatrics..

[18] Quan H, Li B, Couris CM (2011). Updating and validating the charlson comorbidity index and score for risk adjustment in hospital discharge abstracts using data from 6 countries. Am J Epidemiol.

[19] Tsai TC, Orav EJ, Jha AK (2015). Care fragmentation in the postdischarge period surgical readmissions, distance of travel, and postoperative mortality. JAMA Surg.

[20] Austin PC (2009). Using the standardized difference to compare the prevalence of a binary variable between two groups in observational research. Commun Stat - Simul Comput.

[21] Cuzick J (1985). A wilcoxon-type test for trend. Stat Med.

[22] Zou H, Hastie T (2005). Regularization and variable selection via the elastic net. J R Stat Soc Ser B (Statistical Methodol).

[23] Hainmueller J (2011). Entropy balancing for causal effects: a multivariate reweighting method to produce balanced samples in observational studies. Polit Anal.

[24] Royston P, Parmar MKB (2011). The use of restricted mean survival time to estimate the treatment effect in randomized clinical trials when the proportional hazards assumption is in doubt. Stat Med.

[25] Brown KL, Crowe S, Franklin R (2015). Trends in 30-day mortality rate and case mix for paediatric cardiac surgery in the UK between 2000 and 2010. Open Hear..

[26] Abouk R, Grosse SD, Ailes EC, Oster ME (2017). Association of US state implementation of newborn screening policies for critical congenital heart disease with early infant cardiac deaths. JAMA.

[27] Putney AP (2015). Across state lines, a family navigates medical complexity and medicaid hurdles. Health Aff.

[28] Scoggins JF, Fedorenko CR, Donahue SMA, Buchwald D, Blough DK, Ramsey SD (2012). Is Distance to provider a barrier to care for medicaid patients with breast, colorectal, or lung cancer?. J Rural Heal.

[29] Clark CR, Ommerborn MJ, Coull BA, Pham DQ, Haas JS (2016). Income inequities and medicaid expansion are related to racial and ethnic disparities in delayed or forgone care due to cost. Med Care.

[30] Clark CR, Ommerborn MJ, Coull BA, Pham DQ, Haas J (2013). State medicaid eligibility and care delayed because of cost. N Engl J Med.

[31] Miller S, Wherry LR, Kowalski A (2019). Intentional and unintentional effects of safety net programs ‡ four years later: insurance coverage and access to care continue to diverge between aca medicaid expansion and non-expansion states †. AEA Pap Proc.

[32] Cunningham P, Sabik LM, Tehrani AB (2016). Trends in hospital inpatient admissions following early medicaid expansion in California. Med Care Res Rev.

[33] Oster ME, Strickland MJ, Mahle WT (2011). Racial and ethnic disparities in post-operative mortality following congenital heart surgery. J Pediatr.

[34] Nabi J, Tully KH, Cole AP (2020). Access denied: the relationship between patient insurance status and access to high-volume hospitals. Cancer.

[35] Dobson A, DaVanzo J, Haught R, Phap-Hoa L. Comparing the Affordable Care Act’s Financial Impact on Safety-Net Hospitals in States That Expanded Medicaid and Those That Did Not. *Issue Brief (Commonw Fund)*. 2017;2017:1–10. https://europepmc.org/article/med/29232088. Accessed September 1, 2021. 29232088

[36] Williamson CG, Hadaya J, Mandelbaum A (2021). outcomes and resource use associated with acute respiratory failure in safety net hospitals across the United States. Chest.

[37] Sanaiha Y, Rudasill S, Sareh S (2019). Impact of hospital safety-net status on failure to rescue after major cardiac surgery. Surg (United States).

[38] van Velzen C, Clur S, Rijlaarsdam M (2016). Prenatal detection of congenital heart disease—results of a national screening programme. BJOG An Int J Obstet Gynaecol.

[39] Koeckert MS, Ursomanno PA, Williams MR (2017). Reengineering valve patients’ postdischarge management for adapting to bundled payment models. J Thorac Cardiovasc Surg.

[40] Vonlanthen R, Slankamenac K, Breitenstein S (2011). The impact of complications on costs of major surgical procedures: A cost analysis of 1200 patients. Ann Surg.

[41] Lawson EH, Hall BL, Louie R (2013). Association between occurrence of a postoperative complication and readmission: implications for quality improvement and cost savings. Ann Surg.

