# Supplementary material for: Insurance-Based Disparities in Congenital Cardiac Operations in the Era of the Affordable Care Act
Source: Pediatr Cardiol. 2023 Mar 12;44(4):826–35. doi: 10.1007/s00246-023-03136-7 (PMC10063518; doi:10.1007/s00246-023-03136-7)
Supplement: Supplementary file 1 — Supplementary file1 (DOCX 31 KB) [file 246_2023_3136_MOESM1_ESM.docx]

Supplemental Table 1: Multivariable Adjusted Regression Model for Mortality

|  | Adjusted Odds Ratio | | Standard Error | 95% Confidence Interval |
| --- | --- | --- | --- | --- |
| **Age Group** | |  |  |  |
| Neonate | | Reference |  |  |
| Infant | | 0.22 | 0.05 | 0.14, 0.36 |
| 1-3 Years | | 0.11 | 0.03 | 0.07, 0.19 |
| 4-10 Years | | 0.10 | 0.03 | 0.06, 0.17 |
| 11-18 Years | | 0.15 | 0.04 | 0.08, 0.27 |
| Charlson Comorbidity Index (per point) | | 1.76 | 0.09 | 1.60, 1.95 |
| STAT Score (per point) | | 1.33 | 0.03 | 1.27, 1.40 |
| Female Sex | | 1.15 | 0.10 | 0.98, 1.36 |
| Annual Operative Volume (per case) | | 1.00 | 0.00 | 1.00, 1.00 |
| **Procedure** | |  |  |  |
| Glenn Procedure | | Reference |  |  |
| Norwood Procedure | | 0.83 | 0.20 | 0.51, 1.34 |
| Fontan Operation | | 1.43 | 0.44 | 0.78, 2.61 |
| Tetralogy of Fallot Repair | | 0.53 | 0.16 | 0.29, 0.97 |
| Total Anomalous Pulmonary Venous Return Repair | | 1.67 | 0.50 | 0.93, 3.00 |
| Truncus Arteriosus Repair | | 0.93 | 0.42 | 0.38, 2.26 |
| Atrial Septal Defect Repair | | 0.33 | 0.10 | 0.18, 0.60 |
| Ventricular Septal Defect Repair | | 0.39 | 0.10 | 0.24, 0.63 |
| Patent Ductus Arteriosus Repair | | 1.17 | 0.23 | 0.79, 1.73 |
| Arterial Switch | | 0.41 | 0.12 | 0.23, 0.74 |
| Coarctation of Aorta Repair | | 0.54 | 0.15 | 0.31, 0.93 |
| **Calendar Year** | | |  |  |
| 2010 | | Reference |  |  |
| 2011 | | 1.07 | 0.19 | 0.76, 1.52 |
| 2012 | | 0.95 | 0.18 | 0.66, 1.37 |
| 2013 | | 0.74 | 0.14 | 0.52, 1.06 |
| 2014 | | 0.68 | 0.12 | 0.48, 0.97 |
| 2015 | | 0.74 | 0.13 | 0.53, 1.04 |
| 2016 | | 0.93 | 0.16 | 0.67, 1.30 |
| 2017 | | 0.48 | 0.09 | 0.32, 0.70 |
| 2018 | | 0.53 | 0.10 | 0.36, 0.77 |
| **Insurance Status** | | |  |  |
| Private | | Reference |  |  |
| Medicaid | | 1.35 | 0.12 | 1.13, 1.60 |
| _cons | | 0.03 | 0.01 | 0.02, 0.05 |

Supplemental Table 2: Multivariable Adjusted Regression Model for Readmissions

|  | Adjusted Odds Ratio | Standard Error | 95% Confidence Intervals |
| --- | --- | --- | --- |
| **Age Group** |  |  |  |
| Neonate | Reference |  |  |
| Infant | 0.40 | 0.05 | 0.32, 0.50 |
| 1-3 Years | 0.30 | 0.31 | 0.25,0.37 |
| 4-10 Years | 0.25 | 0.02 | 0.21, 0.30 |
| 11-18 Years | 0.22 | 0.03 | 0.17, 0.29 |
| Charlson Comorbidity Index (per point) | 1.23 | 0.07 | 1.10, 1.37 |
| STAT Score (per point) | 1.22 | 0.03 | 1.16, 1.28 |
| Female Sex | 1.17 | 0.09 | 1.00, 1.37 |
| Annual Operative Volume (per case) | 1.00 | 0.00 | 1.00, 1.00 |
| **Procedure** |  |  |  |
| Glenn Procedure | Reference |  |  |
| Norwood Procedure | 1.41 | 0.26 | 0.98, 2.03 |
| Fontan Operation | 3.20 | 0.64 | 2.16, 4.74 |
| Tetralogy of Fallot Repair | 0.61 | 0.12 | 0.42, 0.88 |
| Total Anomalous Pulmonary Venous Return Repair | 0.53 | 0.16 | 0.30, 0.94 |
| Truncus Arteriosus Repair | 0.41 | 0.19 | 0.17, 1.02 |
| Atrial Septal Defect Repair | 0.97 | 0.16 | 0.70, 1.32 |
| Ventricular Septal Defect Repair | 1.19 | 0.42 | 0.59, 2.39 |
| Patent Ductus Arteriosus Repair | 1.68 | 0.33 | 1.15, 2.46 |
| Arterial Switch | 0.80 | 0.18 | 0.52, 1.25 |
| Coarctation of Aorta Repair | 6.64 | 1.40 | 4.38, 10.05 |
| **Calendar Year** | |  |  |
| 2010 | Reference |  |  |
| 2011 | 0.99 | 0.18 | 0.69, 1.43 |
| 2012 | 0.76 | 0.15 | 0.52, 1.11 |
| 2013 | 1.08 | 0.21 | 0.74, 1.57 |
| 2014 | 1.15 | 0.22 | 0.79, 1.67 |
| 2015 | 1.25 | 0.22 | 0.88, 1.77 |
| 2016 | 1.24 | 0.23 | 0.86, 1.77 |
| 2017 | 1.21 | 0.22 | 0.85, 1.72 |
| 2018 | 1.11 | 0.21 | 0.77, 1.60 |
| **Insurance** |  |  |  |
| Private | Reference |  |  |
| Medicaid | 1.12 | 0.06 | 1.01, 1.25 |
| _cons | 0.07 | 0.01 | 0.05, 0.10 |

Supplemental Table 3: Multivariable Adjusted Regression Model for Costs

|  | ß Coefficient | Standard Error | 95% Confidence Interval |  |
| --- | --- | --- | --- | --- |
| **Age Group** |  |  |  | |
| Neonate | Reference |  |  | |
| Infant | -90305 | 6241 | -102558, -78052 | |
| 1-3 Years | -97891 | 5584 | -108855, -86927 | |
| 4-10 Years | -97945 | 6443 | -110596, -85295 | |
| 11-18 Years | -99622 | 6924 | -113217, -86027 | |
| Charlson Comorbidity Index (per point) | 37543 | 4926 | 27872, 47214 | |
| STAT Score (per point) | 7543 | 1212 | 5163, 9923 | |
| Female Sex | -2386 | 3528 | -9312, 4540 | |
| Annual Operative Volume (per case) | -29 | 19 | -66, 9 | |
| **Procedure** |  |  |  | |
| Glenn Procedure | Reference |  |  | |
| Norwood Procedure | 56847 | 14541 | 28296, 85398 | |
| Fontan Operation | 35110 | 8230 | 18952, 51268 | |
| Tetralogy of Fallot Repair | -22902 | 7102 | -36847, -8957 | |
| Total Anomalous Pulmonary Venous Return Repair | 65120 | 24316 | 17377, 112864 | |
| Truncus Arteriosus Repair | 83764 | 24898 | 34878, 132650 | |
| Atrial Septal Defect Repair | 9753 | 8399 | -6738, 26244 | |
| Ventricular Septal Defect Repair | -21104 | 8949 | -38676, -3533 | |
| Patent Ductus Arteriosus Repair | 64068 | 10520 | 43411, 84724 | |
| Arterial Switch | 5695 | 12130 | -18121, 29511 | |
| Coarctation of Aorta Repair | -24844 | 13609 | -51565, 1877 | |
| **Calendar Year** | |  |  | |
| 2010 | Reference |  |  | |
| 2011 | 24529 | 14773 | -4477, 53534 | |
| 2012 | 18408 | 7550 | 3584, 33232 | |
| 2013 | 12512 | 8883 | -4928, 29953 | |
| 2014 | 34005 | 11405 | 11612, 56397 | |
| 2015 | 32476 | 8121 | 16531, 48420 | |
| 2016 | 68100 | 28975 | 11209, 124991 | |
| 2017 | 49824 | 17910 | 14658, 84990 | |
| 2018 | 43996 | 9151 | 26029, 61964 | |
| **Insurance** |  |  |  | |
| Private | Reference |  |  | |
| Medicaid | 16330 | 3989 | 8498, 24163 | |
| _cons | 73441 | 8719 | 56322, 90560 | |

Supplemental Table 4: Entropy-Balanced Multivariable Mortality Model

|  | Adjusted Odds Ratio | | Standard Error | 95% Confidence Interval |
| --- | --- | --- | --- | --- |
| **Age Group** | |  |  |  |
| Neonate | | Reference |  |  |
| Infant | | 0.24 | 0.06 | 0.15, 0.41 |
| 1-3 Years | | 0.12 | 0.04 | 0.06, 0.22 |
| 4-10 Years | | 0.11 | 0.03 | 0.07, 0.18 |
| 11-18 Years | | 0.14 | 0.04 | 0.08, 0.26 |
| Charlson Comorbidity Index (per point) | | 1.76 | 0.10 | 1.58, 1.97 |
| STAT Score (per point) | | 1.33 | 0.04 | 1.26, 1.40 |
| Female Sex | | 1.14 | 0.10 | 0.96, 1.36 |
| Annual Operative Volume (per case) | | 1.00 | 0.00 | 1.00, 1.00 |
| **Procedure** | |  |  |  |
| Glenn Procedure | | Reference |  |  |
| Norwood Procedure | | 0.74 | 0.20 | 0.44, 1.26 |
| Fontan Operation | | 1.24 | 0.45 | 0.61, 2.54 |
| Tetralogy of Fallot Repair | | 0.49 | 0.16 | 0.26, 0.93 |
| Total Anomalous Pulmonary Venous Return Repair | | 1.32 | 0.43 | 0.70, 2.51 |
| Truncus Arteriosus Repair | | 0.99 | 0.48 | 0.38, 2.55 |
| Atrial Septal Defect Repair | | 0.29 | 0.10 | 0.15, 0.56 |
| Ventricular Septal Defect Repair | | 0.33 | 0.09 | 0.19, 0.56 |
| Patent Ductus Arteriosus Repair | | 1.03 | 0.22 | 0.67, 1.57 |
| Arterial Switch | | 0.36 | 0.11 | 0.20, 0.67 |
| Coarctation of Aorta Repair | | 0.47 | 0.14 | 0.26, 0.83 |
| **Calendar Year** | | |  |  |
| 2010 | | Reference |  |  |
| 2011 | | 1.03 | 0.19 | 0.72, 1.48 |
| 2012 | | 0.92 | 0.18 | 0.63, 1.34 |
| 2013 | | 0.72 | 0.14 | 0.50, 1.05 |
| 2014 | | 0.65 | 0.12 | 0.45, 0.94 |
| 2015 | | 0.72 | 0.13 | 0.51, 1.02 |
| 2016 | | 0.90 | 0.16 | 0.64, 1.28 |
| 2017 | | 0.46 | 0.09 | 0.31, 0.68 |
| 2018 | | 0.51 | 0.11 | 0.34, 0.77 |
| **Insurance Status** | | |  |  |
| Private | | Reference |  |  |
| Medicaid | | 1.33 | 0.12 | 1.12, 1.59 |
| _cons | | 0.03 | 0.01 | 0.02, 0.06 |
